# Smartphone sensor accuracy varies from device to device in mobile research: The case of spatial orientation

**DOI:** 10.3758/s13428-020-01404-5

**Published:** 2020-05-29

**Authors:** Tim Kuhlmann, Pablo Garaizar, Ulf-Dietrich Reips

**Affiliations:** 1 Differential Psychology, Assessment & Research Methods, Department of Psychology, University of Siegen, Adolf-Reichwein-Str. 2a, 57068 Siegen, Germany; 2 Research Methods, Assessment, & iScience, Department of Psychology, University of Konstanz, Konstanz, Germany; 3 Faculty of Engineering, University of Deusto, Bilbao, Spain

**Keywords:** Smartphone, Experience sampling, Ambulatory assessment, Sensor data, Tilt, Pitch, Roll

## Abstract

Smartphone usage is increasing around the globe—in daily life and as a research device in behavioral science. Smartphones offer the possibility to gather longitudinal data at little cost to researchers and participants. They provide the option to verify self-report data with data from sensors built into most smartphones. How accurate this sensor data is when gathered via different smartphone devices, e.g., in a typical experience sampling framework, has not been investigated systematically. With the present study, we investigated the accuracy of orientation data about the spatial position of smartphones via a newly invented measurement device, the *RollPitcher*. Objective status of *pitch* (vertical orientation) and *roll* (horizontal orientation) of the smartphone was compared to data gathered from the sensors via web browsers and native apps. Bayesian ANOVAs confirmed that the deviations in pitch and roll differed between smartphone models, with mean inaccuracies per device of up to 2.1° and 6.6°, respectively. The inaccuracies for measurements of roll were higher than for pitch, d = .28, *p* < .001. Our results confirm the presence of heterogeneities when gathering orientation data from different smartphone devices. In most cases, measurement via a web browser was identical to measurement via a native app, but this was not true for all smartphone devices. As a solution to lack of sensor accuracy, we recommend the development and implementation of a coherent research framework and also discuss the implications of the heterogeneities in orientation data for different research designs.

Smartphones present themselves as a powerful tool for researchers. They offer the possibility to gather data from participants in everyday life, largely independent of location and time. In addition, the measurement device is already familiar to participants and causes little to no intrusion or additional costs (Miller, 2012). Smartphones have been widely implemented as part of experience sampling designs (ESMs, e.g., Stieger & Reips, 2019) and in the health sciences (Bert, Giacometti, Gualano, & Siliquini, 2014). In experience sampling designs, smartphones are implemented as tools to gather data from participants at specified times in a diary study or to gather data when events in their lives occur. The topics that can be investigated in real-time include many everyday activities, as most smartphone users carry it around everywhere they go. In addition to subjective measurements, smartphones offer the availability of physical sensors that are already integrated and easily accessible (Miller, 2012). These include, among others, GPS, Bluetooth, and data on spatial orientation. Data from these sensors can be gathered via apps and browsers and the advantages of using these data are more and more evident in the behavioral, social, and health sciences. In the health sector, the data are used to recognize physical activity, mostly using data from the accelerometer. Studies have shown that smartphones are capable of achieving similar accuracies for physical activity recognition as dedicated devices, such as smart watches and heart rate monitors (Brajdic & Harle, 2013; Case et al., 2015). Sensor data can also be used to identify falling or other medical emergencies (Yavuz et al., 2010) and to improve accessibility for wheelchair users (Gupta, Holloway, Heravi, & Hailes, 2015). The Bluetooth sensor has been implemented to detect whether a person is in a work or social situation (Lathia, Pejovic, Rachuri, Mascolo, Musolesi, & Rentfrow, 2013). In the social sciences, studies have further been conducted that link a person's well-being to their surroundings via GPS (MacKerron & Mourato, 2013; Stieger & Reips, 2019). Stieger and Reips (2019) investigated data from both smartphone sensors and from open-access Internet databases on temperature, longitude, latitude, altitude, wind speed, rainfall, and further environment-based variables to predict fluctuations of well-being by using a smartphone-based mobile experience sampling method. In their study, they found a high correlation between smartphone GPS measurement of altitudes and Google Maps measurement of altitudes, but a consistent difference in absolute measurement.

In order for the sensor data to be useful to researchers, it has to be accurate and a valid indicator for the behavior. If the data from the implemented devices shows a large amount of error, conclusions drawn from the data are necessarily unreliable. This fact is much more important in the context of smartphone studies as compared to previous research studies where the measurement device was given out by researchers to the participants (Miller, 2012). Previous studies allowed researchers to pick an adequate device, preprogram all the necessary parts, and check the reliability of the data. Error might still be present, but it can be investigated and potentially mitigated. Furthermore, it is largely homogenous across the sample. In most smartphone studies, participants are using their own device and only download an app or open their web browser to participate. This presents researchers with additional problems, as there may be a large heterogeneity of data across devices. Naturally, over the Internet it is impossible to check all devices for their idiosyncrasies (Reips, Buchanan, Krantz, & McGraw, 2015).

With regard to data from the objective sensors, this problem is rather new to behavioral and social scientists. Data on the devices’ location and orientation telling us indirectly about fine-grained motion behaviors of a large number of people have only been introduced with the development of smartphones. Their implementation has for the most part been focused on games and interactive apps. The implementation of sensor data in behavioral and social science research is only beginning, meaning the requirements for accuracy of measurement via user devices are not well investigated. Blunck et al. (2013) developed a taxonomy of heterogeneities and their sources in mobile phones. The focus of the present study is heterogeneities due to the device, i.e., resulting from the platform, hardware, and OS. Thus, here we are investigating a special case of using heterogeneous consumer-grade equipment in Internet-based research.

## Gathering a device’s orientation from sensors

Social and behavioral researchers run their experiments on mobile devices using native and web apps. Native apps run on top of the operating system of the device and use compiled code (i.e., Java/Kotlin for Android devices, Objective-C/Swift for iOS devices). Web apps run within a web browser (Google Chrome, Apple Safari, Mozilla Firefox) and use web APIs (Application Programming Interfaces) through JavaScript code. Moreover, there are some application development frameworks (Xamarin, Appcelerator, Adobe PhoneGap) that are able to port their code to multiple platforms such as Android through different approaches. Embedding a web browser within a native app to run JavaScript code is a common strategy for these cross-platform frameworks. Therefore, we have to know how an app was developed to categorize it as native or web app.

The distinction between native and web apps is important when we are gathering information from mobile device’s sensors. Native applications are able to collect data from hardware sensors directly while web applications are unable to do so, for security reasons. However, most native mobile applications do not take values directly from sensors but use what mobile operating systems call "software sensors". Software sensors provide estimates of actual position, orientation, and motion values by combining the readings of various hardware sensors such as accelerometers, gyroscopes, magnetometers, or barometers. Hardware sensors in today’s smartphones are similar to circuit chips in appearance and work electronically.

Using software sensors is a good development strategy because it allows developers to forget about the peculiarities of each hardware sensor and delegate the integration of their values to the mobile operating system. Considering this is the approach followed by mobile web browsers, the differences between native and web apps should be minimal.

The focus of the current study is sensing the smartphone’s spatial orientation. Data from the orientation software sensor is used to take photo sphere images or when playing games that use the tilt of the phone as input. In the behavioral sciences, this data has been implemented as a proxy measurement for body posture (Kuhlmann & Reips, 2020) and position of wheelchairs (Milenković et al., 2013). In future studies, possible implementations include the measurement of motor tasks in experience sampling designs or the measurement of the environment by placing the phone in the surrounding environment. Questionnaire items or tasks could also be answered by tilting the phone instead of responding on a typical scale.

**Fig. 1:**
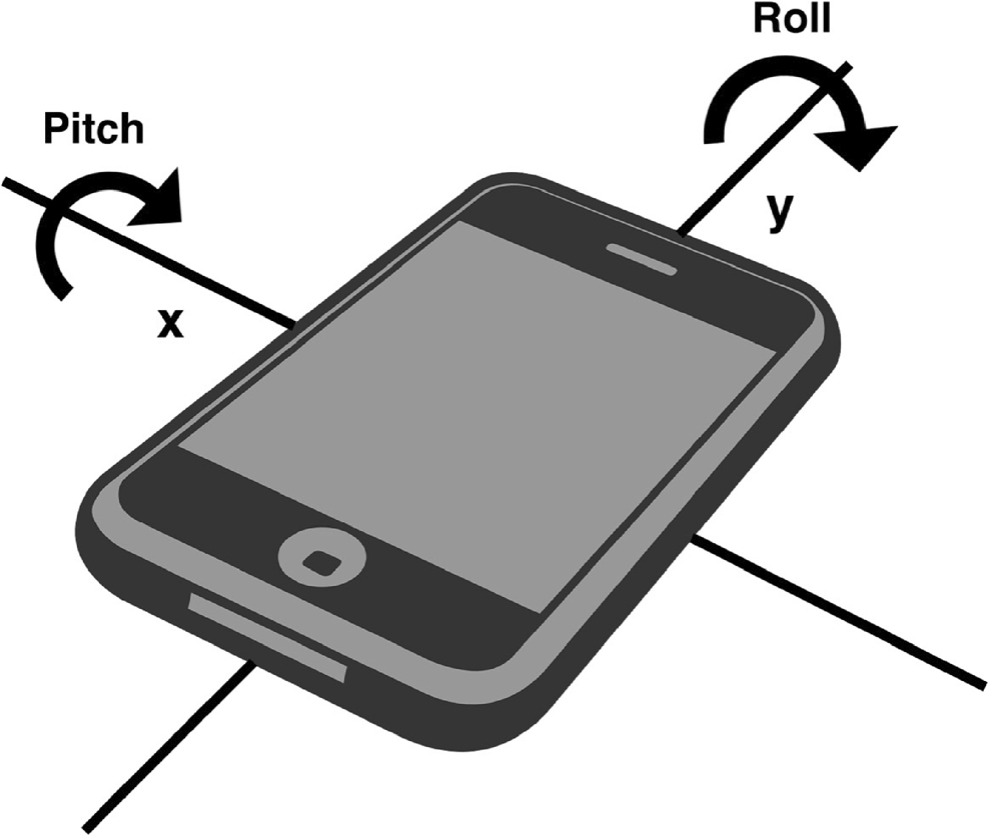
Illustration of orientation measures *pitch* and *roll* in relation to smartphone axes

On-board technology provides information about the tilt of the smartphone across three different axes, *x*, *y*, and *z*. The rotation around one of these axes, *z*, indicates the cardinal direction of the phone. The other two rotations describe the rotation of the device itself around the other two axes (see Fig. 1). As mentioned before, data about the location of the smartphone is gathered by using a software sensor that integrates information from the accelerometer, gyroscope, and magnetometer of the device to provide acceleration data from gravity. If the device is lying on a flat surface, this force is aiming to the ground, at a 90° angle to the screen and the two axes around which pitch and roll are measured. No force is present along the axes that are parallel to the long and short side of the phone. When the device is tilted around its axes, the force is no longer vertical to the screen. This deviation is used to calculate the values for pitch and roll, indicating the tilt of the smartphone in relation to its flat position.

As mentioned before, orientation data is gathered via different APIs and sensor frameworks. Native mobile apps can gather orientation data via sensor-specific frameworks for different operating systems (Blunck et al., 2013). Web apps use the DeviceOrientation Event specification (Tibbet & Volodine, 2017) to access to this data. The different APIs and frameworks present a potential source of heterogeneity between different software implementations and devices. How the actual values of orientation are computed already differs depending on the implemented browser and operating system (Deveria, 2018).

## Accuracy of smartphone sensor data

The accuracy of other sensor data has been investigated in previous studies. Stisen and colleagues (2015) investigated the heterogeneities of data gathered from *accelerometers* of different smartphone and smartwatch models. The acceleration sensor measures the acceleration of the devices along different axes and provides useful data to distinguish different activities. The authors were interested in the effect of possible heterogeneities on activity recognition due to different sensors, devices, and workload. In their study, they found that heterogeneities impaired the performance of human activity recognition. The impairments differed significantly between different devices, as sensor data accuracy varied between different models and manufacturers. For some investigated devices, deviations from the actual acceleration were as large as the difference between standing still and accelerating on a fast train (Stisen et al., 2015). They also found some indications of heterogeneities for data from the orientation sensor on activity recognition, but this was not the main part of their investigation, as orientation data is not the best choice for this task.

Data on the accuracy of the spatial orientation is mostly based on the investigation of external influences on the accuracy and *natural drifts* in values, mostly implemented during production (Grewal & Andrews, 2010). One of these external influences is the temperature at which the orientation sensor operates. Changing temperature results in inaccuracies of the readings (Weinberg, 2011). As this inaccuracy is predictable and quite consistent, most orientation sensors are coupled with a temperature sensor. Another source of inaccuracies is acceleration and vibration. This is especially a problem for compact orientation sensors without much buffer that are implemented in mobile devices (Weinberg, 2011). The orientation sensor itself cannot be calibrated via a simple user prompt as is the case for the cardinal direction. Some studies did implement calibration techniques involving external sensors and expensive setups (e.g., Umek & Kos, 2016).

It has not been investigated so far how the implementation via different applications and frameworks influences the orientation data, e.g., whether the data is gathered via a browser or a native app. As mentioned in the previous section, frameworks and browsers read and transform data on tilt differently (Deveria, 2018; Tibbet & Volodine, 2017). In addition, app development frameworks might perform transformations of the data that suit the intended implementation of the target audience. For example, applications developed via the MIT App Inventor transform the values of pitch and roll when they cross 90° of tilt (MIT App Inventor Public Open Source, 2018).

The current study investigates the accuracy of orientation data in implementations that closely resemble those of actual study designs. We gather data from smartphones that are participants’ actual phones without modifying their settings, installed apps, preferred browser, etc. To ascertain the real values of pitch and roll that the smartphone is rotated to, we designed and built a mounting device for smartphones, *RollPitcher*, which allows for the independent manipulation of pitch and roll in a controlled lab setting. Our study therefore fills an important gap in knowledge between accuracy measurements of sensors close to production and their accuracy in actual implementations in smartphone studies. Our hypotheses are:

- H1: The accuracy of the orientation data differs between smartphone devices
- H2: The accuracy of the orientation data differs, to a small degree, between modes of measurement on the same smartphone
- H3: The inaccuracies of the orientation data are consistent across measurements of different angles of the same smartphone with the same software, i.e., deviations from real values correlate across measurements

## Method

### RollPitcher, the smartphone mounting device

**Fig. 2:**
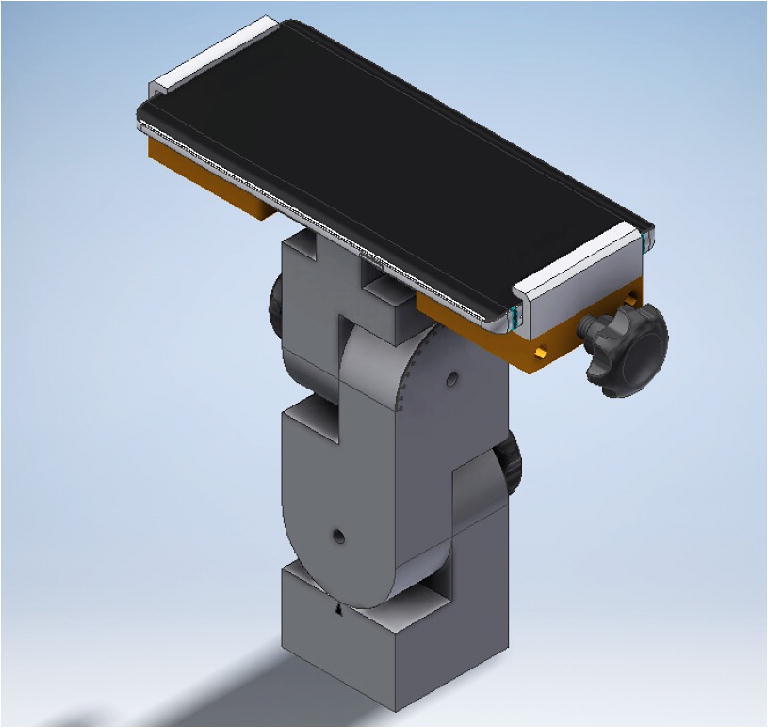
Technical drawing of RollPitcher, the smartphone mounting device

Two RollPitcher devices were custom built by the scientific workshop of the University of Konstanz, one made of metal and one entirely out of plastic, except for small parts. They consist of a solid base, on top of which the mount was attached. A technical drawing of the mounts is shown in Fig. 2. They have two different hinges, which allows for the separate adjustment of pitch and roll values. The mount for the smartphone itself is made out of solid plastic in both RollPitchers and has a cut-out in the base to allow all smartphone models to lie flat on the surface despite possible bumps from cameras on the backside.

**Fig. 3:**
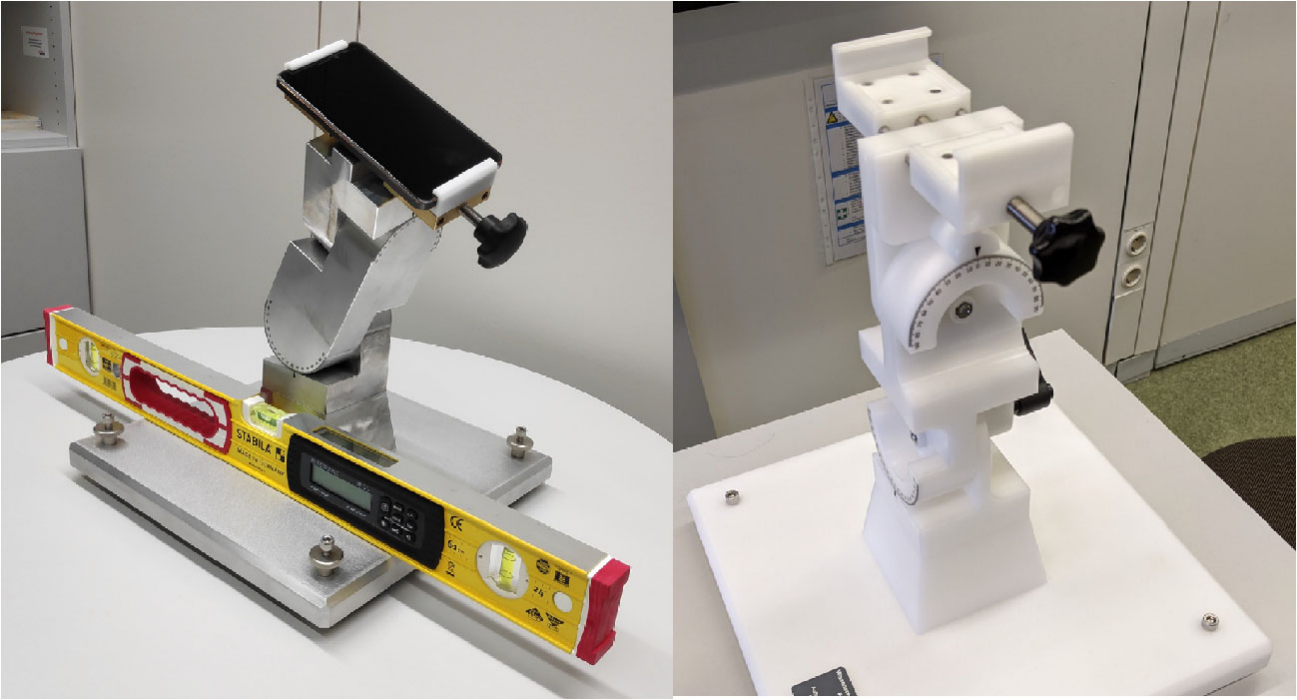
RollPitcher metal mounting device with smartphone and mechanic’s level (***left***) and plastic mounting device (***right***)

The actual metal and plastic devices weigh about 8 and 3 kg, respectively. The metal and plastic devices are shown in Fig. 3. The base can be adjusted via four different screws and thus allows leveling of the base precisely to 0°. Levelling out the base was achieved by adjusting the position with a high-precision digital mechanic’s level, the *Stabila STB196E-2-60P*, with a maximum error of .05° at 0°. To ascertain the precision of the objective angle positions, the mechanic’s level was used on some occasions to measure the pitch and roll of the smartphone by placing it on top of the screen along both axes. These values were within 0.15° of the proposed pitch and roll values, confirming the precision of the mount and procedure.

### Sample of smartphones

A total of 56 different smartphones were measured, 31 Android devices, 24 iOS devices, and one Windows 10 device. All devices were measured via the browser implementation. In addition, a subsample of 39 devices was also measured via the native apps. A complete list of the smartphone models with their OS is shown in Table 1. The smartphones were selected to represent a typical sample of smartphones implemented in the research setting during a smartphone study. We used smartphones from participants who took part for course credit or remuneration. The phones cover different manufacturers, models, and operating systems. We did not alter any of the settings, OS updates or installed browsers to follow the logic of simulating a real study situation as close as possible, apart from the objective position of the device.

**Table 1:** Measured smartphone models

Manufacturer	Model	Number of devices tested	Operating system
Samsung	A5	1	Android
Samsung	Duos	1	Android
Samsung	J5	1	Android
Samsung	S4	1	Android
Samsung	S5	1	Android
Samsung	S6	1	Android
Samsung	S7	2	Android
Samsung	S8	12	Android
Samsung	S9+	1	Android
Huawei	P8	1	Android
Huawei	P8 Lite	3	Android
Huawei	P9 Lite	3	Android
Huawei	P20 Lite	1	Android
Xiaomi	Pocophone F1	1	Android
Apple	iPhone 5	1	iOS
Apple	iPhone 6	9	iOS
Apple	iPhone 6s	2	iOS
Apple	iPhone SE	2	iOS
Apple	iPhone 7	6	iOS
Apple	iPhone 8	1	iOS
Apple	iPhone XR	1	iOS
CAT	S61	1	Android
Nokia	Lumia 950	1	Windows 10 Mobile
Honor	9 Lite	1	Android
Motorola	G4+	1	Android

### Measures

The values of pitch and roll were gathered via two different software implementations, a website and native applications in Android and iOS. The measurement on a website was implemented via the DeviceOrientation Event Specification (Tibbet & Volodine, 2018). This specification provides several DOM events related to the orientation and motion of a device. The *deviceorientation* event supplies the physical orientation of the device, the *devicemotion* event supplies the acceleration of the device, and the *compassneedscalibration* event is used to warn web apps about the need of recalibration of the compass being used to provide data for one of the other two events. Considering this, we created a simple web app, available in the OSF archive, and registered it to receive *deviceorientation* events. The events provide four attributes, of which two are of interest to the present study (see Fig. 1). The pitch of the device is represented by *beta.* It describes the top-down orientation around the *x*-axis, represented in degrees with values ranging from – 180 to 180. The roll is represented by *gamma*, which describes the left–right orientation around the *y*-axis, represented in degrees with values ranging from – 90 to 90. The code to register *deviceorientation* events via the web app is the following:

window.addEventListener("deviceorientation", function(event) {

// process event.beta and event.gamma

}, true);

The beta angle is 0° when the device's top and bottom are the same distance from the earth's surface. If the device is in a vertical plane and the top of the screen pointing upwards, the value of beta is 90°. The gamma angle is 0° when the device's left and right sides are the same distance from the surface of the earth.

We developed the native application for Android ourselves following the guidelines provided by the Android official documentation. Although the orientation sensor was deprecated in Android 2.2 (API level 8), the Android sensor framework provides alternate methods for acquiring device orientation. The orientation angles are computed by using a device's geomagnetic field sensor in combination with the device's accelerometer. The use of these two hardware sensors provides three orientation angles, two of which are relevant for the present study: *pitch* describes the degrees of rotation about the *x*-axis, i.e., top-bottom tilt from – 180 to 180 degrees; *roll* describes the degrees of rotation about the *y*-axis, i.e., left-right tilt from – 90 to 90 degrees. The angles correspond to the aforementioned beta and gamma values from the Device Orientation API. The native application implemented on iOS devices was the sensor reading app “Sensors Multitool”, available free of charge from the Apple AppStore. It provides separate sensor readings for *pitch* and *roll*, named *x* and *y*, and displays them on-screen.

### Procedure

At the start of the measurements, the native apps were installed on participants’ smartphones and their screen was set to portrait mode. Before the smartphone was mounted, any protective case was removed and RollPitcher was levelled out to a precision of ± .05°. The smartphone was then located in position. The measurements took place according to a scheduled sequence of angle combinations. The pitch angles had the values 0°, 30°, 60°, and 85° in both directions. Roll angles were 0°, 15°, and 30° in both directions. These values were chosen to represent *typical* locations of smartphones during everyday use. The pitch values typically deviate more from the null point than roll values (Kuhlmann & Reips, 2020). The vertical angle of 90° was not measured, as for this angle there is no meaningful value of roll because the smartphone is standing on the side. Every combination of pitch and roll angles was implemented via the mounting device and the data sent three times, with a pause of 1–2 s between sending, to a Firebase database. This data had a precision of nine decimal points. Data from the iOS native app was recorded by hand to a spreadsheet with a precision of one decimal point. This procedure led to 35 different combinations of angles measured per device, once via native app, and once via web browser. The browser was chosen based on participants’ standard settings for web browsing, representing the most likely option that a participant would partake in an Internet-based questionnaire.

### Statistical analyses

The data was imported from Firebase and spreadsheets into R. Analyses were carried out in R and JASP (JASP Team, 2018). Data files, R scripts and the browser app are available at https://osf.io/hfcx8/. The three repeated measures of pitch and roll for each angle combination were close to identical, differing by less than .01° on average. The arithmetic mean of the three measurements was used in statistical analyses.

## Results

First, we describe the exclusion of suspect roll values. Then the descriptive statistics for deviations of pitch and roll are presented and thereafter the statistical analyses comparing different smartphone devices.

### Exclusion of roll values at pitch of 85°

Roll values that were gathered at pitch angles of 85° were excluded from all combined analyses as these showed unusually large deviations from the objective values. The deviations from objective roll values ranged from – 28.5° to 55.7° (SD = 9.9°) at pitch angles of 85°. The deviations from objective roll values for all other pitch angles was – 11.6° to 10.0° (SD = 1.8°). Possible explanations and interpretations for this qualitative difference are reviewed in the Discussion section. The main hypothesis is that the angle of 85° is too close to 90°, at which there are no meaningful values for roll.

### The influence of RollPitcher building material

We conducted measurements via two RollPitchers that differed in the material they were made of, from metal and the other from plastic. To assess whether the material of the RollPitcher has an influence on the measurement, we performed the same measurement routine on identical smartphones in RollPitchers in short succession. This was carried out with four different smartphones. A Bayesian repeated measures ANOVA with RollPitcher device as the repeated measures variable and pitch deviation as the dependent variable was calculated. The Bayes factor was BF_10_ = .156, providing no evidence for an effect of the device, but moderate evidence in favor of no difference. The effect size of the repeated measures factor was η^2^ = .0004, indicating that less than 0.1% of the variance in pitch deviations could be attributed to the RollPitcher device. The results for roll deviation were similar with BF_10_ = .136 and η^2^ < .001.

### Deviations of pitch and roll from objective values

**Fig. 4:**
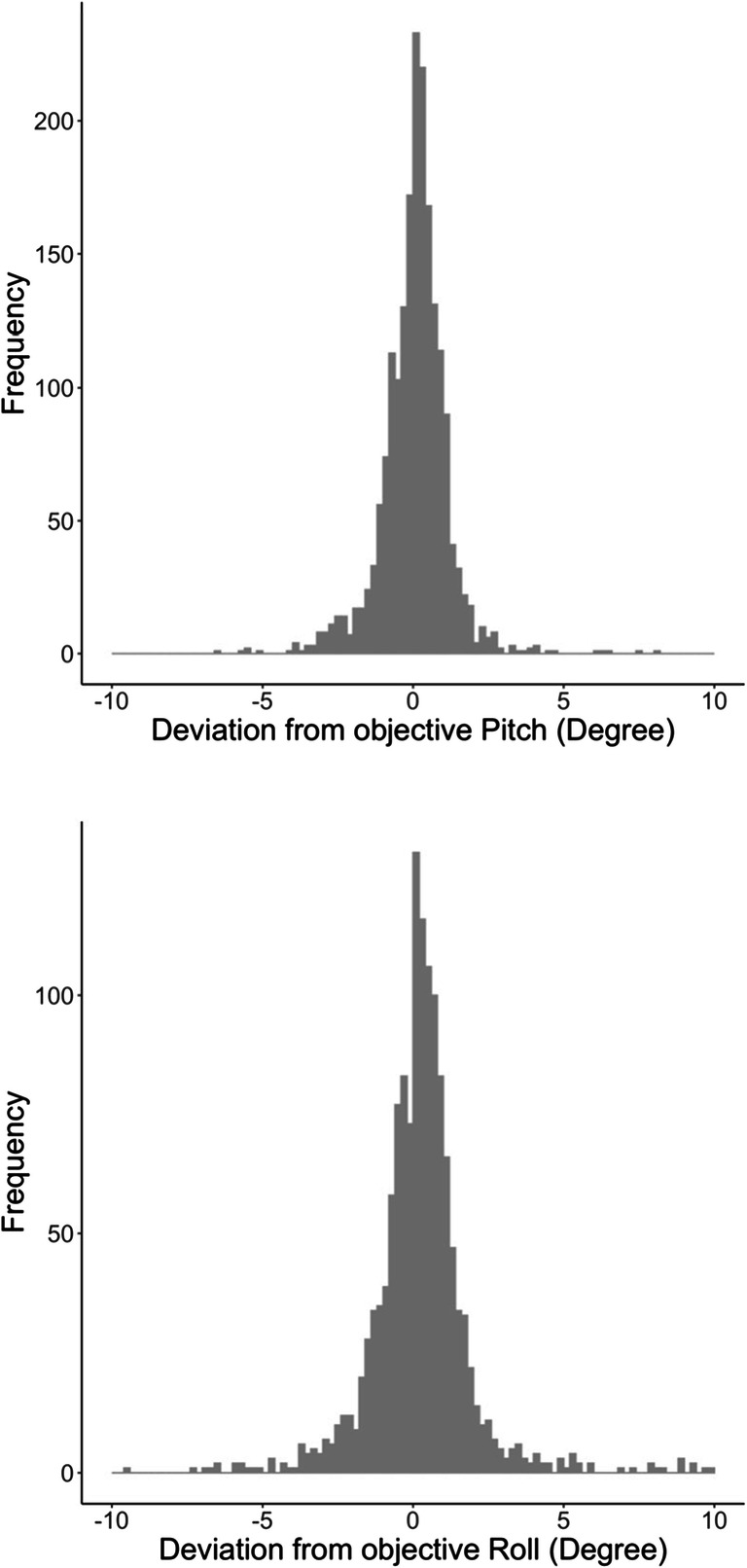
Browser-measured deviations of the sensor gathered pitch values (*top*) and roll values (*bottom*) from the objective position of the smartphones across all devices

The distributions of the deviation of sensor measured pitch and roll values from the objective angles are shown in Fig. 4. The distributions are based on data gathered via the browser of the smartphones. The mean deviation was 0.05° for pitch, ranging from – 17.8° to 8.1° (SD = 1.2°). For the roll values, the mean deviation was 0.20°, ranging from – 11.6° to 10.0° (SD = 1.8°).

**Fig. 5:**
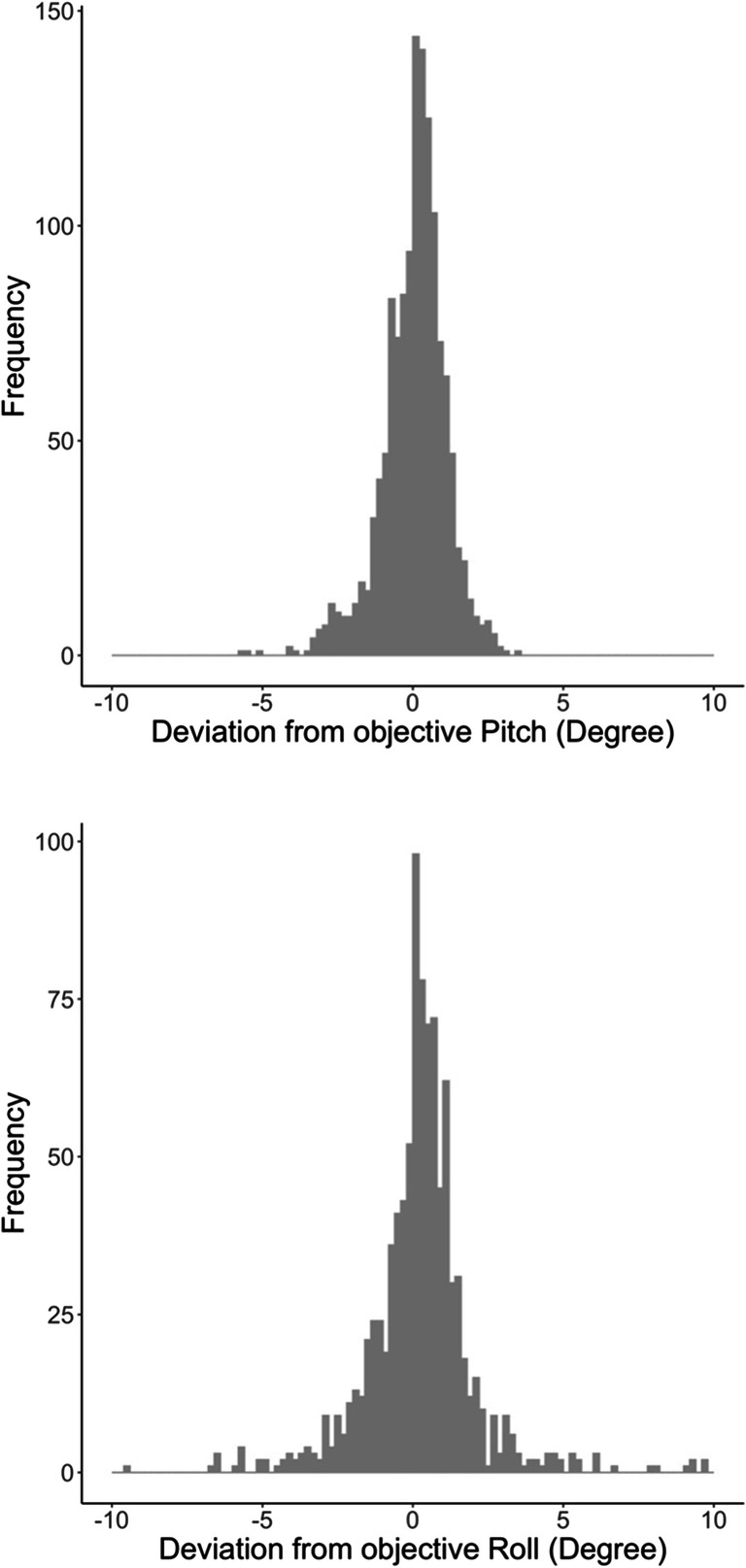
Native app-measured deviations of the sensor gathered pitch values (*top*) and roll values (*bottom*) from the objective position of the smartphones across all devices

The distributions based on data from the native apps are shown in Fig. 5. The distributions are similar to the ones gathered via the browser. The mean deviation was 0.05° for pitch, ranging from – 5.71° to 3.48° (SD = 1.1°). For the roll values, the mean deviation was 0.21°, ranging from – 14.7° to 10.3° (SD = 2.0°).

The correlation between the values from the browser and the native app was r = .91 for pitch and r = .90 for roll. Overall, the results show a high, albeit not perfect, overlap between the two modes of measurement.

### Comparison of devices and mode of measurement

Bayesian repeated measures ANOVAs for the absolute deviations of pitch and roll values from the objective angles were calculated. This allows for a comparison of the deviation in both directions and removes the possibility of inaccuracies in both directions to cancel each other out. It therefore allows for a better comparison of the heterogeneity between devices and software. The repeated measures factor was mode of measurement, i.e., native app or web browser. The 56 smartphone devices were included as a between factor. Results for the deviation values of pitch are shown in Table 2.

**Table 2:** Bayesian repeated measures ANOVA of absolute pitch deviations

Models	BF _10_	Error %
Null model	1.000	
Mode of measurement (repeated)	75.81	3.248
Smartphone device	1.32 * 10^103^	0.195

**Fig. 6:**
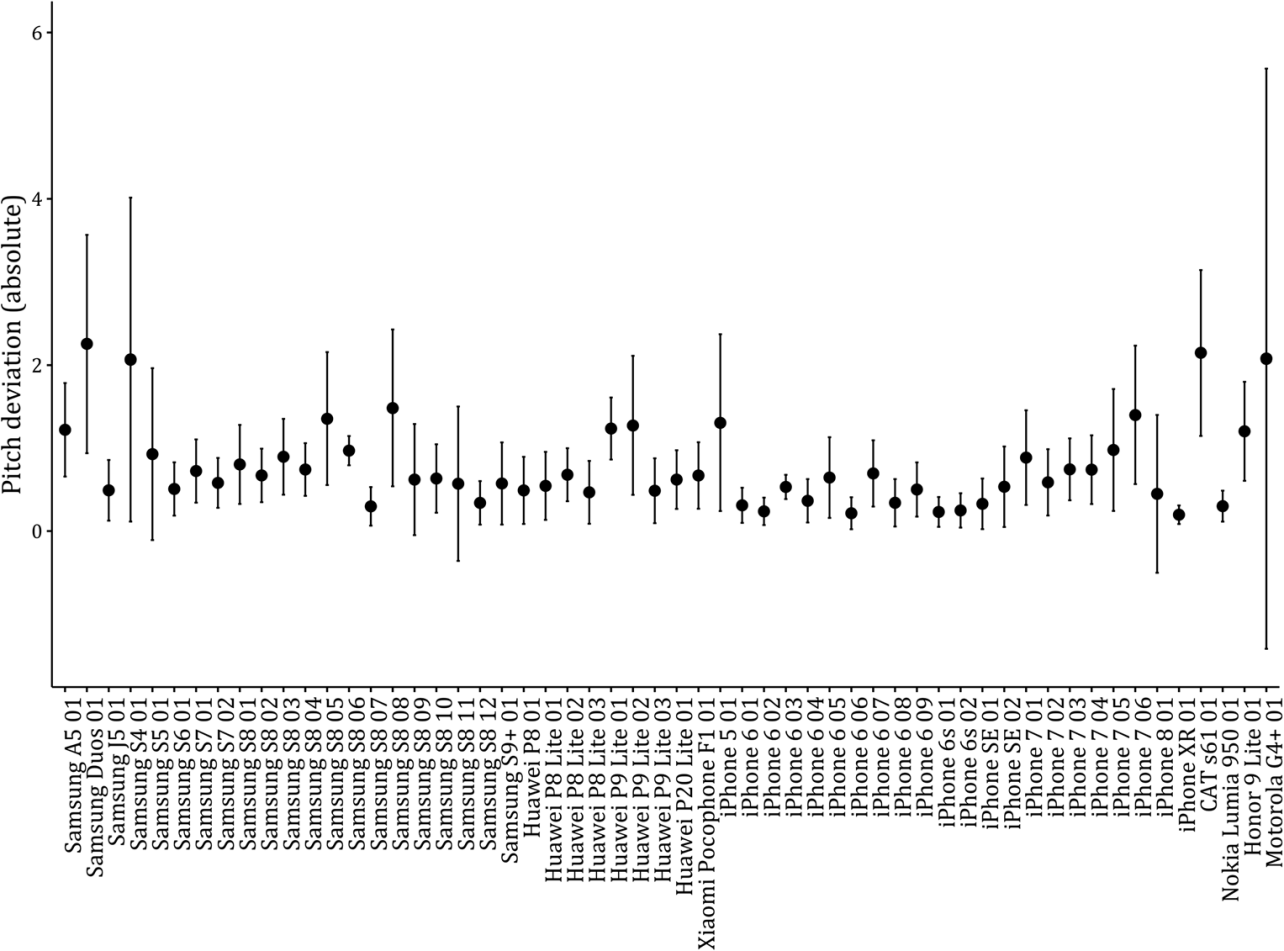
Mean browser-based absolute deviations from the objective pitch values and their SDs by smartphone device

The heterogeneities in pitch deviations due to smartphone device did show very strong support for an influence of the smartphone device, with a Bayes factor of BF_10_ = 1.32 * 10^103^. The explained variance in pitch deviations by smartphone device amounted to η^2^ = .38. Also, the Bayes factor for the repeated measures supports a difference of pitch deviations due to mode of measurement, BF_10_ = 75.81, but the explained variance was very small with η^2^= .001. When including the smartphone device in the null model and computing the Bayes factors for adding the interaction it showed very strong support for improving the model, BF_10_ = 9.64 * 10^9^. This result signifies that the mode of measurement, browser vs. native app, did not affect all devices equally, with some devices showing larger differences than others. The mean absolute deviations and their standard deviations for the browser values of pitch are shown in Fig. 6.

Results for the Bayesian repeated measures ANOVA of deviation values of roll are shown in Table 3. The results for roll are consistent to the ones for pitch. The Bayes factor for the repeated measures did provide evidence against a difference of roll deviations due to mode of measurement, BF_10_ = 0.08. The explained variance was very low with η^2^ < .001. The heterogeneities in roll deviations due to smartphone device did show very strong support for an influence of the smartphone device with a Bayes factor of BF_10_ = 1.73 * 10^136^. The explained variance in roll deviations by smartphone device was higher as compared to pitch deviations, η^2^= .57.

**Table 3:** Bayesian repeated measures ANOVA of absolute roll deviations

Models	BF _10_	Error %
Null model	1.000	
Mode of measurement (repeated)	0.08	1.042
Smartphone device	1.73 * 10^136^	0.212

**Fig. 7:**
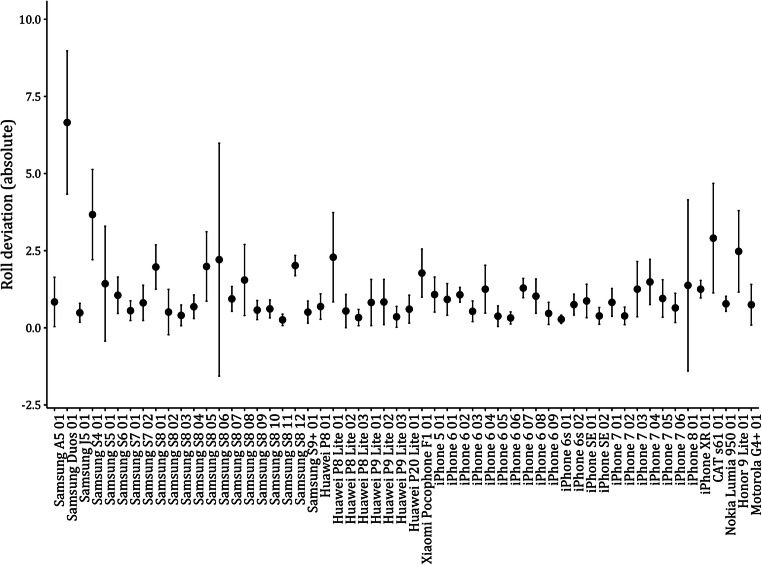
Mean browser-based absolute deviations from the objective roll values and their SDs by smartphone device

When including the smartphone device in the null model and computing the Bayes factors for adding the mode of measurement and the interaction, the main effect for mode of measurement did not improve the model, BF_10_ = 0.08, but the interaction again did, BF_10_ = 4.23 * 10^13^. This signifies that for some smartphone devices, the mode of measurement does change the values, but not as a main effect. The mean absolute deviations and their standard deviations for the browser values of roll are shown in Fig. 7.

The following analyses are only reported for the browser-based measurements because the differences between browser and native measurements were very small and the browser data was available for all devices.

A linear mixed model with smartphone device as a random effect was calculated to compare the deviations in pitch values to the deviations in roll values. The analysis confirmed the impression from the descriptive plots. Deviations from objective roll values were higher than the deviations from pitch values by an average of 0.36°, t (3260) = 10.91, *p* < .001, d = .28.

Hypothesis 3, the consistency of the deviations within the same smartphone device, was tested via ICCs. We were interested in the consistency of the deviations across the different objective angles that were measured. For the pitch values, the inaccuracies did show a moderate amount of consistency within devices, ICC = .26, *p* < .001. This signifies that pitch measurement deviations within a device were somewhat consistent across measurement occasions. For the roll values, the consistency of inaccuracies within devices was smaller, ICC = .07, *p* < .001. Roll measurement deviations were not as stable within the measured devices.

### Comparison of operating systems and manufacturer

**Fig. 8:**
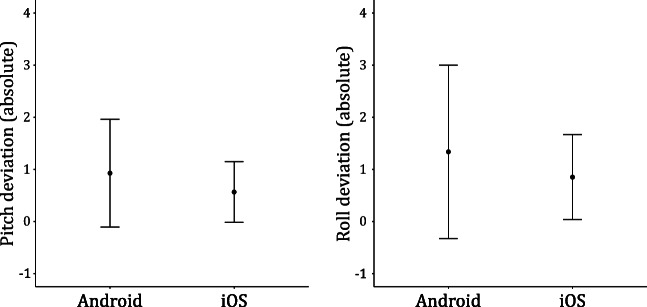
Absolute browser-based deviations of pitch and roll aggregated for operating systems

To compare the impact of the operating system and the manufacturers of the device on the accuracy of measurement, linear mixed models with random intercepts for each device were calculated. The dependent variable was always the absolute tilt deviation and the angle, pitch, or roll, was included as a covariate. As there was only one device with a different OS than Android or iOS, the Nokia Lumia 950, the analysis compared only these two operating systems. The OS of the device did show an association with the accuracy of measurement, t(52.94) = – 2.39, *p* = .021. iOS devices showed slightly smaller inaccuracies, but the effect size was very small with η^2^= .03. The mean inaccuracy for pitch and roll of both operating systems is shown in Fig. 8.

The manufacturer of the device, e.g., Samsung, Apple, Huawei, did not predict inaccuracies in pitch or roll deviation, t(53.95) = – 0.174, *p* = .86.

## Discussion

Our results show that heterogeneities in pitch and roll data are present for the orientation sensor. Devices differ in accuracy with some showing mean deviations close to 0° and little variance while other devices show mean inaccuracies of up to 2°, on some occasions reaching over 6° compared to the objective tilt. The deviations are higher for measurements of roll than they are for measurements of pitch. Hypothesis 1, referring to the heterogeneities between devices, was therefore supported. The results are in line with findings on the accelerometer (Stisen et al., 2015).

Whether a browser or a native application was used to gather data did not have an influence on the measurement accuracy of the sensor data, overall. However, there was a significant interaction between smartphone device and mode of measurement, pointing towards some differences between devices. For some devices, the values of pitch and roll were basically identical, regardless of whether they were measured via a web browser or native application. For other devices, the differences were more pronounced. In addition to these heterogeneities, the directions, e.g., signs of angles, did differ depending on the software. Depending on the planned study, a reversal of angles in some devices does have the potential to seriously alter the results of analyses. The results with regard to mode of measurement partially support our second hypothesis. The software implementation does have an influence, but to a smaller degree than the differences across devices. This is in line with previous suggestions (Blunck et al., 2013) and technical considerations when implementing software sensors (Deveria, 2018).

The magnitude of the deviations is not negligible, but their importance depends on the research question that is investigated. If the orientation sensor is merely supposed to indicate switches between portrait and landscape mode or capture falling behavior, small heterogeneities might not be as impactful. In one of our studies, however, deviations of 4–5° from the actual values are close in magnitude to the effect size when trying to measure body posture (Kuhlmann & Reips, 2020).

The analyses on differences in inaccuracies between different operating systems and manufacturers only revealed small or non-significant effects. The inaccuracies of specific devices contribute more variance than distinguishing by operating system or manufacturer.

For the present study, we excluded roll values that were acquired at 85° of pitch because of their inaccuracy. If this correction is not performed, the heterogeneities and inaccuracies are magnitudes higher to the point of approaching random values of roll. In the Procedure section, we mentioned that at pitch values of 90°, there does not exist a meaningful value for roll because the device is standing on the side. Tilt around the *y*-axis, i.e., roll, is always at a right angle to the gravitational force and different roll values can therefore not be distinguished. Our results suggest that this problem is present at pitch angles lower than 90°. This explains the wide range of deviations measured at pitch angles of 85°. Future research should determine the exact angle from which the qualitative difference occurs. Our advice to researchers is to handle roll data at pitch angles approaching 90° with care and, whenever possible, check for unusually large variance or deviations. Another, more complex, solution is to use the raw data from the sensors and calculate quaternions instead of Euler angles (Favre, Jolles, Siegrist, & Aminian, 2006).

Our results indicate that inaccuracies are moderately consistent within devices, meaning that a deviation in pitch in one direction at a certain angle does show a positive correlation with the deviation in pitch at other angles. This supports our third hypothesis, at least for deviations in pitch. When conducting longitudinal studies, this is possibly an important factor as variables are often ipsatized, i.e. centered around the person mean, in these designs to separate between- and within-person effects (Curran & Bauer, 2011). Ipsatizing creates variables for the within-person effect that are centered around a person mean. Stable deviations within one device mean that these ipsatized values are influenced to a lesser degree by heterogeneities and deviations of orientation data. The effect is not completely removed because the correlations within a device are not perfect and vary across devices and tilt. It is still an important fact to consider when evaluating whether and how big of a problem heterogeneities are in the context in a given research design.

Comparisons between persons, i.e., devices, are influenced to a higher degree. Not only are the inaccuracies a bigger problem because they are not consistent across devices, but the possibility of different software implementations also opens the possibility of more profound problems for the comparability (Blunck et al., 2013). There is no binding standard on the signage of pitch and roll, meaning that a negative pitch for one software solution might be the same value with opposite sign in others. There is no way to ascertain comparability of signs apart from testing them beforehand. Assuming that the number of software implementations is not too large, this should not be too problematic. A bit more problematic is the possibility that certain values are transformed or cut off by the software. For example, the MIT App inventor transforms values with an absolute value of over 90° to either roll back to 0 with increasing tilt or it freezes them at the angle until the value gets lower again (MIT App Inventor Public Open Source, 2018). Transformations of data are usually automated with certain applications in mind, e.g., games, which may not be in line with researchers’ interests. Furthermore, these transformations are not easy to find in manuals, as they pertain to a very specific topic not usually of interest to everyday app developers. A recommendation for developers of research-oriented frameworks is to provide a coherent API where researchers can forget about device particularities and get similar values in cross-platform setups. Such an API would make comparisons easier for researchers and solve many of the problems of comparability before they arise in data analysis.

### Limitations

The present study is limited by the number of devices that were investigated. Though they were selected to be comparable to the situation in a typical smartphone study, they do not cover the complete range of possible devices in a research design. This is not the aim of the present study, though. We want to investigate whether there exists a problem when implementing the orientation sensor and provide researchers with an estimate of possible effects. Our study finds there is a problem and the effects are substantial.

Another limitation is the value of the angles of pitch and roll that we investigated. They do not cover the entire range of possible angles, but merely represent a typical combination that reflects common pitch and roll values. Our study is not guaranteed to also reflect extreme cases, e.g., turning the phone upside down, or reflect every other possible combination of angles. We do provide a meaningful number of combinations of pitch and roll, however, that covers the spectrum of a typical smartphone study (Kuhlmann & Reips, 2020).

## Conclusions

The present study does show that heterogeneities are present in data on the spatial orientation of smartphones. The inaccuracies are usually between 0.5 and 3° (except for 85° pitch angle), large enough to potentially influence results. They differ depending on the smartphone device. Future studies could aim to expand the number of devices tested and possibly a database could be created as a reference for researchers. A database would not allow researchers to perfectly adjust their design and analyses, but might provide helpful data and guidelines when implementing orientation data in a study. It might also help other researchers to estimate the stability of their findings. The results of the present study do provide an estimate of the magnitude of heterogeneity across different smartphones and research designs in which they matter.
